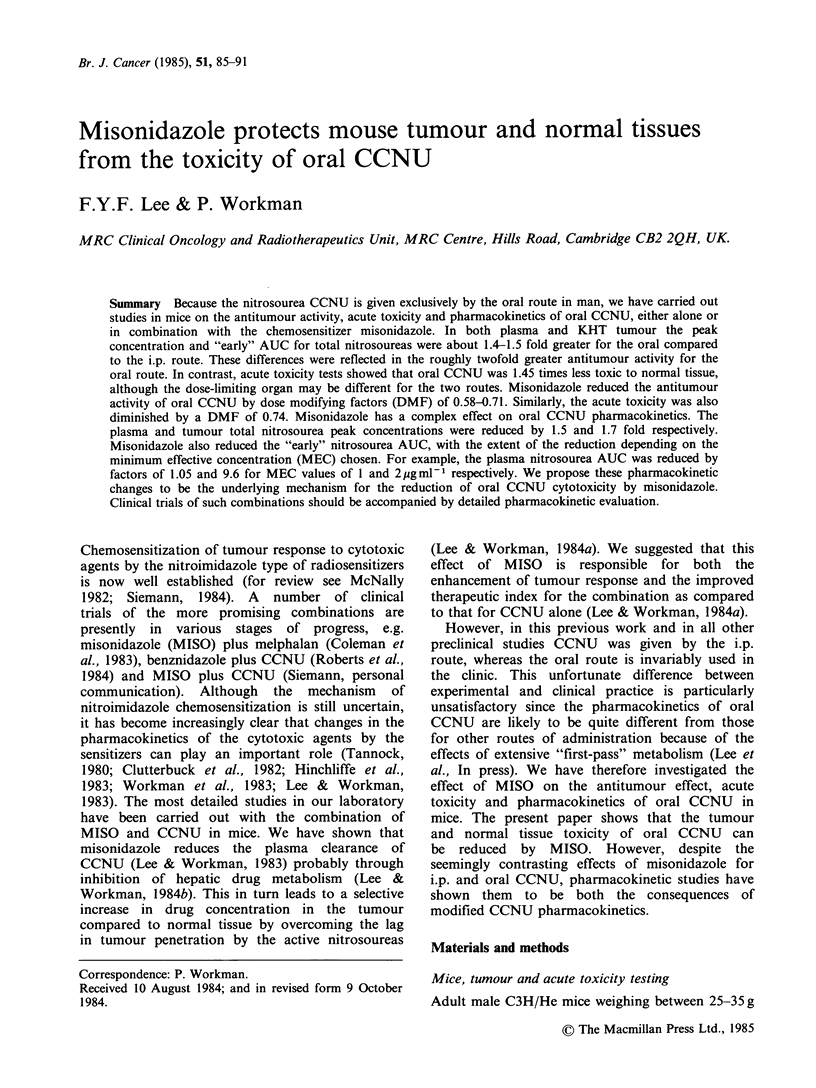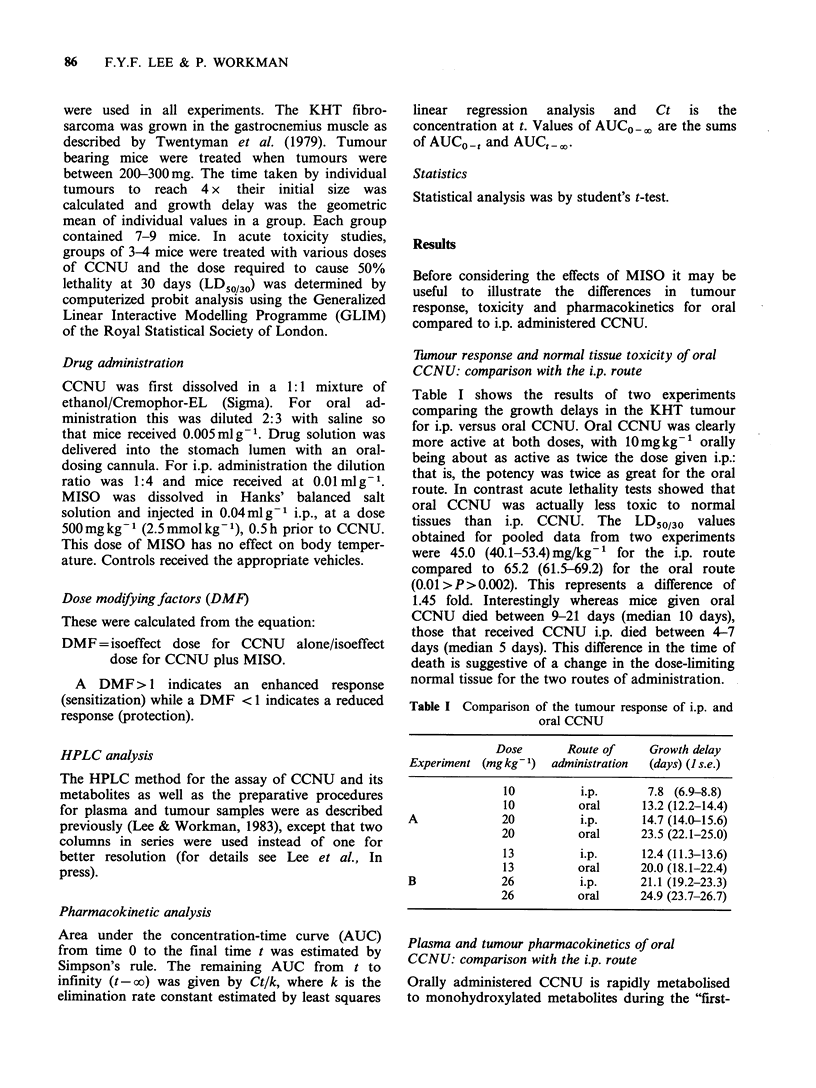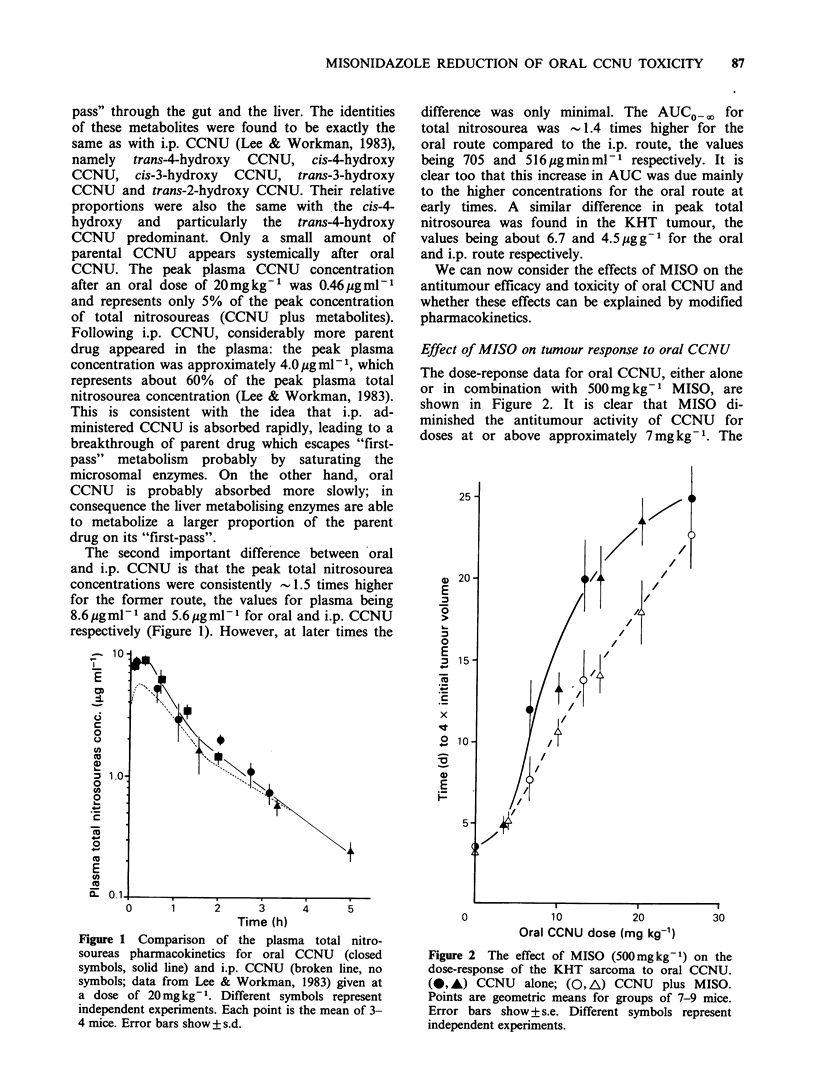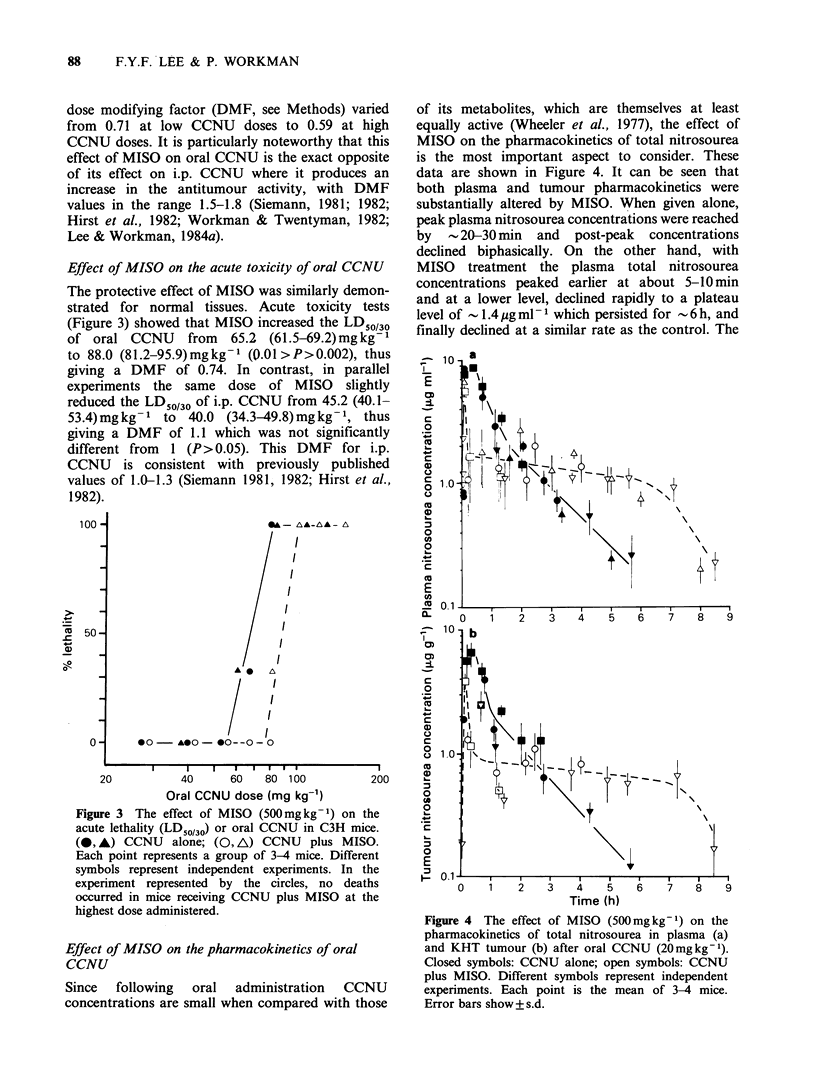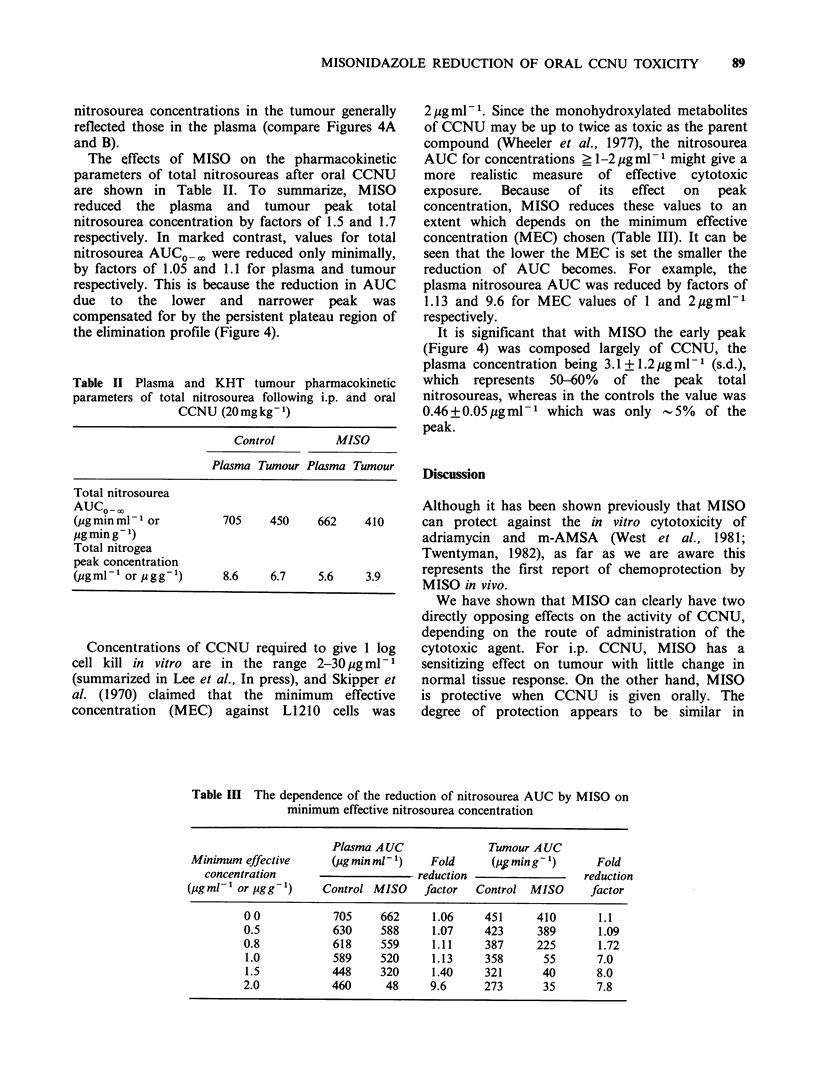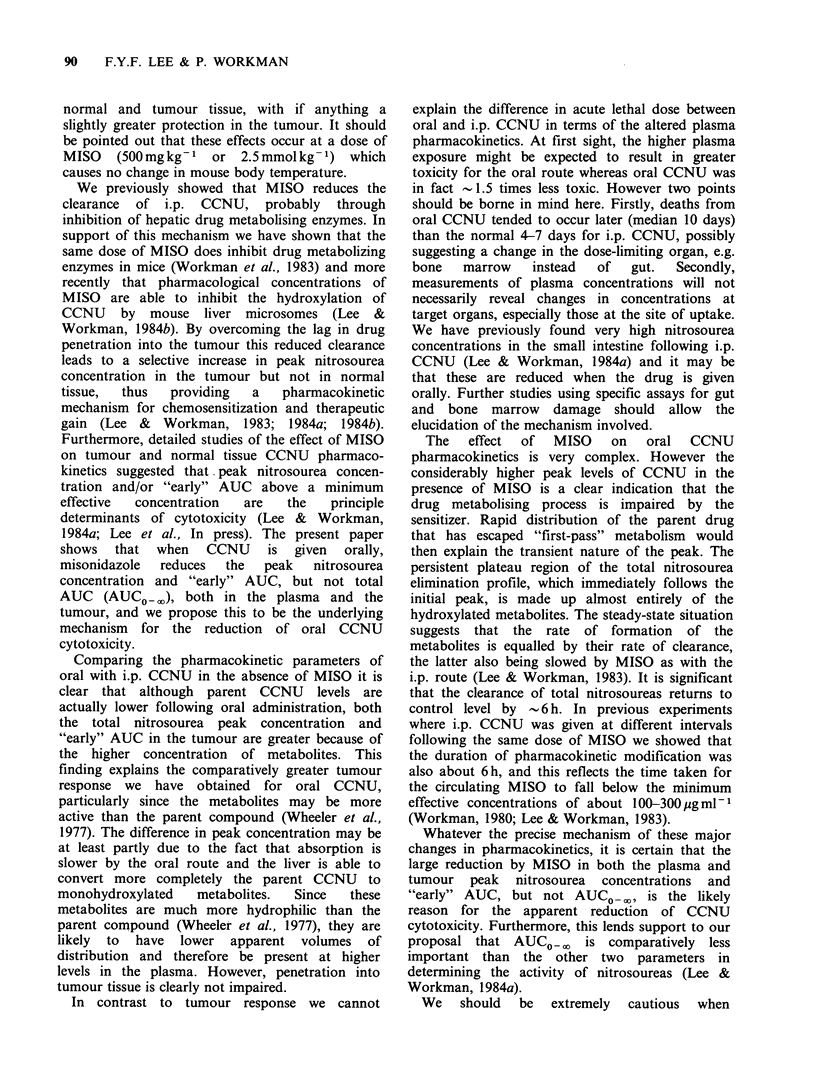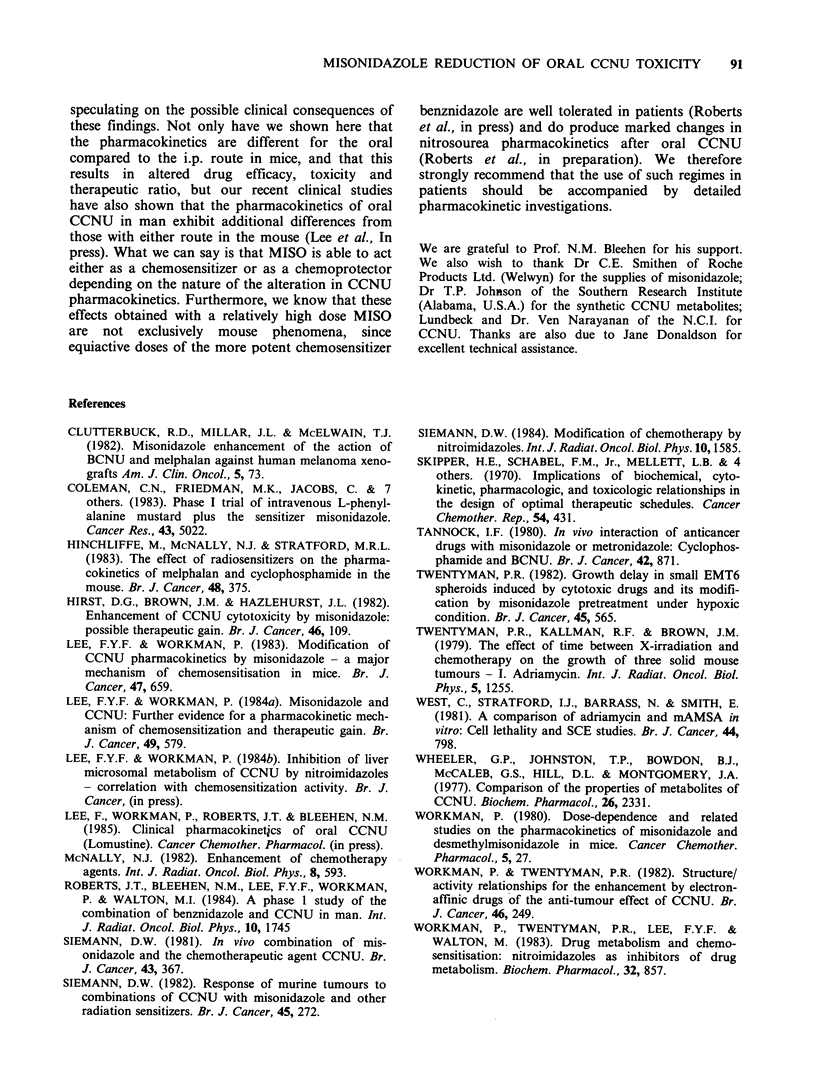# Misonidazole protects mouse tumour and normal tissues from the toxicity of oral CCNU.

**DOI:** 10.1038/bjc.1985.12

**Published:** 1985-01

**Authors:** F. Y. Lee, P. Workman

## Abstract

Because the nitrosourea CCNU is given exclusively by the oral route in man, we have carried out studies in mice on the antitumour activity, acute toxicity and pharmacokinetics of oral CCNU, either alone or in combination with the chemosensitizer misonidazole. In both plasma and KHT tumour the peak concentration and "early" AUC for total nitrosoureas were about 1.4-1.5 fold greater for the oral compared to the i.p. route. These differences were reflected in the roughly twofold greater antitumour activity for the oral route. In contrast, acute toxicity tests showed that oral CCNU was 1.45 times less toxic to normal tissue, although the dose-limiting organ may be different for the two routes. Misonidazole reduced the antitumour activity of oral CCNU by dose modifying factors (DMF) of 0.58-0.71. Similarly, the acute toxicity was also diminished by a DMF of 0.74. Misonidazole has a complex effect on oral CCNU pharmacokinetics. The plasma and tumour total nitrosourea peak concentrations were reduced by 1.5 and 1.7 fold respectively. Misonidazole also reduced the "early" nitrosourea AUC, with the extent of the reduction depending on the minimum effective concentration (MEC) chosen. For example, the plasma nitrosourea AUC was reduced by factors of 1.05 and 9.6 for MEC values of 1 and 2 micrograms ml-1 respectively. We propose these pharmacokinetic changes to be the underlying mechanism for the reduction of oral CCNU cytotoxicity by misonidazole. Clinical trials of such combinations should be accompanied by detailed pharmacokinetic evaluation.


Br. J. Cancer (1985), 51, 85–91

### Misonidazole protects mouse tumour and normal tissues from the toxicity of oral CCNU

F.Y.F. Lee & P. Workman

*MRC Clinical Oncology and Radiotherapeutics Unit, MRC Centre, Hills Road, Cambridge CB2 2QH, UK.*

**Summary** Because the nitrosourea CCNU is given exclusively by the oral route in man, we have carried out studies in mice on the antitumour activity, acute toxicity and pharmacokinetics of oral CCNU, either alone or in combination with the chemosensitizer misonidazole. In both plasma and KHT tumour the peak concentration and "early" AUC for total nitrosoureas were about 1.4–1.5 fold greater for the oral compared to the i.p. route. These differences were reflected in the roughly twofold greater antitumour activity for the oral route. In contrast, acute toxicity tests showed that oral CCNU was 1.45 times less toxic to normal tissue, although the dose-limiting organ may be different for the two routes. Misonidazole reduced the antitumour activity of oral CCNU by dose modifying factors (DMF) of 0.58–0.71. Similarly, the acute toxicity was also diminished by a DMF of 0.74. Misonidazole has a complex effect on oral CCNU pharmacokinetics. The plasma and tumour total nitrosourea peak concentrations were reduced by 1.5 and 1.7 fold respectively. Misonidazole also reduced the "early" nitrosourea AUC, with the extent of the reduction depending on the minimum effective concentration (MEC) chosen. For example, the plasma nitrosourea AUC was reduced by factors of 1.05 and 9.6 for MEC values of 1 and $2 \mu g\,ml^{-1}$ respectively. We propose these pharmacokinetic changes to be the underlying mechanism for the reduction of oral CCNU cytotoxicity by misonidazole. Clinical trials of such combinations should be accompanied by detailed pharmacokinetic evaluation.

Chemosensitization of tumour response to cytotoxic agents by the nitroimidazole type of radiosensitizers is now well established (for review see McNally 1982; Siemann, 1984). A number of clinical trials of the more promising combinations are presently in various stages of progress, e.g. misonidazole (MISO) plus melphalan (Coleman *et al.*, 1983), benznidazole plus CCNU (Roberts *et al.*, 1984) and MISO plus CCNU (Siemann, personal communication). Although the mechanism of nitroimidazole chemosensitization is still uncertain, it has become increasingly clear that changes in the pharmacokinetics of the cytotoxic agents by the sensitizers can play an important role (Tannock, 1980; Clutterbuck *et al.*, 1982; Hinchliffe *et al.*, 1983; Workman *et al.*, 1983; Lee & Workman, 1983). The most detailed studies in our laboratory have been carried out with the combination of MISO and CCNU in mice. We have shown that misonidazole reduces the plasma clearance of CCNU (Lee & Workman, 1983) probably through inhibition of hepatic drug metabolism (Lee & Workman, 1984*b*). This in turn leads to a selective increase in drug concentration in the tumour compared to normal tissue by overcoming the lag in tumour penetration by the active nitrosoureas (Lee & Workman, 1984*a*). We suggested that this effect of MISO is responsible for both the enhancement of tumour response and the improved therapeutic index for the combination as compared to that for CCNU alone (Lee & Workman, 1984*a*).

However, in this previous work and in all other preclinical studies CCNU was given by the i.p. route, whereas the oral route is invariably used in the clinic. This unfortunate difference between experimental and clinical practice is particularly unsatisfactory since the pharmacokinetics of oral CCNU are likely to be quite different from those for other routes of administration because of the effects of extensive "first-pass" metabolism (Lee *et al.*, In press). We have therefore investigated the effect of MISO on the antitumour effect, acute toxicity and pharmacokinetics of oral CCNU in mice. The present paper shows that the tumour and normal tissue toxicity of oral CCNU can be reduced by MISO. However, despite the seemingly contrasting effects of misonidazole for i.p. and oral CCNU, pharmacokinetic studies have shown them to be both the consequences of modified CCNU pharmacokinetics.

Correspondence: P. Workman.
Received 10 August 1984; and in revised form 9 October 1984.

### Materials and methods

### *Mice, tumour and acute toxicity testing*

Adult male C3H/He mice weighing between 25–35 g

© The Macmillan Press Ltd., 1985

were used in all experiments. The KHT fibrosarcoma was grown in the gastrocnemius muscle as described by Twentyman *et al.* (1979). Tumour bearing mice were treated when tumours were between 200–300 mg. The time taken by individual tumours to reach 4× their initial size was calculated and growth delay was the geometric mean of individual values in a group. Each group contained 7–9 mice. In acute toxicity studies, groups of 3–4 mice were treated with various doses of CCNU and the dose required to cause 50% lethality at 30 days ($LD_{50/30}$) was determined by computerized probit analysis using the Generalized Linear Interactive Modelling Programme (GLIM) of the Royal Statistical Society of London.

*Drug administration*

CCNU was first dissolved in a 1:1 mixture of ethanol/Cremophor-EL (Sigma). For oral administration this was diluted 2:3 with saline so that mice received $0.005\,ml\,g^{-1}$. Drug solution was delivered into the stomach lumen with an oral-dosing cannula. For i.p. administration the dilution ratio was 1:4 and mice received at $0.01\,ml\,g^{-1}$. MISO was dissolved in Hanks' balanced salt solution and injected in $0.04\,ml\,g^{-1}$ i.p., at a dose $500\,mg\,kg^{-1}$ ($2.5\,mmol\,kg^{-1}$), 0.5 h prior to CCNU. This dose of MISO has no effect on body temperature. Controls received the appropriate vehicles.

*Dose modifying factors (DMF)*

These were calculated from the equation:

DMF = isoeffect dose for CCNU alone/isoeffect dose for CCNU plus MISO.

A DMF > 1 indicates an enhanced response (sensitization) while a DMF < 1 indicates a reduced response (protection).

*HPLC analysis*

The HPLC method for the assay of CCNU and its metabolites as well as the preparative procedures for plasma and tumour samples were as described previously (Lee & Workman, 1983), except that two columns in series were used instead of one for better resolution (for details see Lee *et al.*, In press).

*Pharmacokinetic analysis*

Area under the concentration-time curve (AUC) from time 0 to the final time $t$ was estimated by Simpson's rule. The remaining AUC from $t$ to infinity ($t-\infty$) was given by $Ct/k$, where $k$ is the elimination rate constant estimated by least squares linear regression analysis and $Ct$ is the concentration at $t$. Values of $AUC_{0-\infty}$ are the sums of $AUC_{0-t}$ and $AUC_{t-\infty}$.

*Statistics*

Statistical analysis was by student's $t$-test.

### Results

Before considering the effects of MISO it may be useful to illustrate the differences in tumour response, toxicity and pharmacokinetics for oral compared to i.p. administered CCNU.

*Tumour response and normal tissue toxicity of oral CCNU: comparison with the i.p. route*

Table I shows the results of two experiments comparing the growth delays in the KHT tumour for i.p. versus oral CCNU. Oral CCNU was clearly more active at both doses, with $10\,mg\,kg^{-1}$ orally being about as active as twice the dose given i.p.: that is, the potency was twice as great for the oral route. In contrast acute lethality tests showed that oral CCNU was actually less toxic to normal tissues than i.p. CCNU. The $LD_{50/30}$ values obtained for pooled data from two experiments were 45.0 (40.1–53.4) $mg/kg^{-1}$ for the i.p. route compared to 65.2 (61.5–69.2) for the oral route ($0.01 > P > 0.002$). This represents a difference of 1.45 fold. Interestingly whereas mice given oral CCNU died between 9–21 days (median 10 days), those that received CCNU i.p. died between 4–7 days (median 5 days). This difference in the time of death is suggestive of a change in the dose-limiting normal tissue for the two routes of administration.

**Table I** Comparison of the tumour response of i.p. and oral CCNU

| *Experiment* | *Dose* ($mg\,kg^{-1}$) | *Route of administration* | *Growth delay (days) (1 s.e.)* |
|---|---|---|---|
| | 10 | i.p. | 7.8 (6.9–8.8) |
| | 10 | oral | 13.2 (12.2–14.4) |
| A | 20 | i.p. | 14.7 (14.0–15.6) |
| | 20 | oral | 23.5 (22.1–25.0) |
| | 13 | i.p. | 12.4 (11.3–13.6) |
| | 13 | oral | 20.0 (18.1–22.4) |
| B | 26 | i.p. | 21.1 (19.2–23.3) |
| | 26 | oral | 24.9 (23.7–26.7) |

*Plasma and tumour pharmacokinetics of oral CCNU: comparison with the i.p. route*

Orally administered CCNU is rapidly metabolised to monohydroxylated metabolites during the "firstpass" through the gut and the liver. The identities of these metabolites were found to be exactly the same as with i.p. CCNU (Lee & Workman, 1983), namely *trans*-4-hydroxy CCNU, *cis*-4-hydroxy CCNU, *cis*-3-hydroxy CCNU, *trans*-3-hydroxy CCNU and *trans*-2-hydroxy CCNU. Their relative proportions were also the same with the *cis*-4-hydroxy and particularly the *trans*-4-hydroxy CCNU predominant. Only a small amount of parental CCNU appears systemically after oral CCNU. The peak plasma CCNU concentration after an oral dose of $20\,mg\,kg^{-1}$ was $0.46\,\mu g\,ml^{-1}$ and represents only 5% of the peak concentration of total nitrosoureas (CCNU plus metabolites). Following i.p. CCNU, considerably more parent drug appeared in the plasma: the peak plasma concentration was approximately $4.0\,\mu g\,ml^{-1}$, which represents about 60% of the peak plasma total nitrosourea concentration (Lee & Workman, 1983). This is consistent with the idea that i.p. administered CCNU is absorbed rapidly, leading to a breakthrough of parent drug which escapes "first-pass" metabolism probably by saturating the microsomal enzymes. On the other hand, oral CCNU is probably absorbed more slowly; in consequence the liver metabolising enzymes are able to metabolize a larger proportion of the parent drug on its "first-pass".

The second important difference between oral and i.p. CCNU is that the peak total nitrosourea concentrations were consistently ~1.5 times higher for the former route, the values for plasma being $8.6\,\mu g\,ml^{-1}$ and $5.6\,\mu g\,ml^{-1}$ for oral and i.p. CCNU respectively (Figure 1). However, at later times the difference was only minimal. The $AUC_{0-\infty}$ for total nitrosourea was ~1.4 times higher for the oral route compared to the i.p. route, the values being 705 and $516\,\mu g\,min\,ml^{-1}$ respectively. It is clear too that this increase in AUC was due mainly to the higher concentrations for the oral route at early times. A similar difference in peak total nitrosourea was found in the KHT tumour, the values being about 6.7 and $4.5\,\mu g\,g^{-1}$ for the oral and i.p. route respectively.

We can now consider the effects of MISO on the antitumour efficacy and toxicity of oral CCNU and whether these effects can be explained by modified pharmacokinetics.

*Effect of MISO on tumour response to oral CCNU*

The dose-reponse data for oral CCNU, either alone or in combination with $500\,mg\,kg^{-1}$ MISO, are shown in Figure 2. It is clear that MISO diminished the antitumour activity of CCNU for doses at or above approximately $7\,mg\,kg^{-1}$. The

**Figure 1** Comparison of the plasma total nitrosoureas pharmacokinetics for oral CCNU (closed symbols, solid line) and i.p. CCNU (broken line, no symbols; data from Lee & Workman, 1983) given at a dose of $20\,mg\,kg^{-1}$. Different symbols represent independent experiments. Each point is the mean of 3–4 mice. Error bars show ± s.d.

**Figure 2** The effect of MISO ($500\,mg\,kg^{-1}$) on the dose-response of the KHT sarcoma to oral CCNU. (●, ▲) CCNU alone; (○, △) CCNU plus MISO. Points are geometric means for groups of 7–9 mice. Error bars show ± s.e. Different symbols represent independent experiments.

dose modifying factor (DMF, see Methods) varied from 0.71 at low CCNU doses to 0.59 at high CCNU doses. It is particularly noteworthy that this effect of MISO on oral CCNU is the exact opposite of its effect on i.p. CCNU where it produces an increase in the antitumour activity, with DMF values in the range 1.5–1.8 (Siemann, 1981; 1982; Hirst *et al.*, 1982; Workman & Twentyman, 1982; Lee & Workman, 1984*a*).

### *Effect of MISO on the acute toxicity of oral CCNU*

The protective effect of MISO was similarly demonstrated for normal tissues. Acute toxicity tests (Figure 3) showed that MISO increased the $LD_{50/30}$ of oral CCNU from 65.2 (61.5–69.2) mg kg$^{-1}$ to 88.0 (81.2–95.9) mg kg$^{-1}$ ($0.01 > P > 0.002$), thus giving a DMF of 0.74. In contrast, in parallel experiments the same dose of MISO slightly reduced the $LD_{50/30}$ of i.p. CCNU from 45.2 (40.1–53.4) mg kg$^{-1}$ to 40.0 (34.3–49.8) mg kg$^{-1}$, thus giving a DMF of 1.1 which was not significantly different from 1 ($P > 0.05$). This DMF for i.p. CCNU is consistent with previously published values of 1.0–1.3 (Siemann 1981, 1982; Hirst *et al.*, 1982).

**Figure 3** The effect of MISO (500 mg kg$^{-1}$) on the acute lethality ($LD_{50/30}$) or oral CCNU in C3H mice. (●, ▲) CCNU alone; (○, △) CCNU plus MISO. Each point represents a group of 3–4 mice. Different symbols represent independent experiments. In the experiment represented by the circles, no deaths occurred in mice receiving CCNU plus MISO at the highest dose administered.

### *Effect of MISO on the pharmacokinetics of oral CCNU*

Since following oral administration CCNU concentrations are small when compared with those of its metabolites, which are themselves at least equally active (Wheeler *et al.*, 1977), the effect of MISO on the pharmacokinetics of total nitrosourea is the most important aspect to consider. These data are shown in Figure 4. It can be seen that both plasma and tumour pharmacokinetics were substantially altered by MISO. When given alone, peak plasma nitrosourea concentrations were reached by ~20–30 min and post-peak concentrations declined biphasically. On the other hand, with MISO treatment the plasma total nitrosourea concentrations peaked earlier at about 5–10 min and at a lower level, declined rapidly to a plateau level of ~1.4 μg ml$^{-1}$ which persisted for ~6 h, and finally declined at a similar rate as the control. The

**Figure 4** The effect of MISO (500 mg kg$^{-1}$) on the pharmacokinetics of total nitrosourea in plasma (a) and KHT tumour (b) after oral CCNU (20 mg kg$^{-1}$). Closed symbols: CCNU alone; open symbols: CCNU plus MISO. Different symbols represent independent experiments. Each point is the mean of 3–4 mice. Error bars show ± s.d.

nitrosourea concentrations in the tumour generally reflected those in the plasma (compare Figures 4A and B).

The effects of MISO on the pharmacokinetic parameters of total nitrosoureas after oral CCNU are shown in Table II. To summarize, MISO reduced the plasma and tumour peak total nitrosourea concentration by factors of 1.5 and 1.7 respectively. In marked contrast, values for total nitrosourea $AUC_{0-\infty}$ were reduced only minimally, by factors of 1.05 and 1.1 for plasma and tumour respectively. This is because the reduction in AUC due to the lower and narrower peak was compensated for by the persistent plateau region of the elimination profile (Figure 4).

**Table II** Plasma and KHT tumour pharmacokinetic parameters of total nitrosourea following i.p. and oral CCNU ($20\,mg\,kg^{-1}$)

| | *Control* | | *MISO* | |
|---|---|---|---|---|
| | *Plasma* | *Tumour* | *Plasma* | *Tumour* |
| Total nitrosourea $AUC_{0-\infty}$ ($\mu g\,min\,ml^{-1}$ or $\mu g\,min\,g^{-1}$) | 705 | 450 | 662 | 410 |
| Total nitrogea peak concentration ($\mu g\,ml^{-1}$ or $\mu g\,g^{-1}$) | 8.6 | 6.7 | 5.6 | 3.9 |

Concentrations of CCNU required to give 1 log cell kill *in vitro* are in the range $2–30\,\mu g\,ml^{-1}$ (summarized in Lee *et al.*, In press), and Skipper *et al.* (1970) claimed that the minimum effective concentration (MEC) against L1210 cells was $2\,\mu g\,ml^{-1}$. Since the monohydroxylated metabolites of CCNU may be up to twice as toxic as the parent compound (Wheeler *et al.*, 1977), the nitrosourea AUC for concentrations $\geq 1–2\,\mu g\,ml^{-1}$ might give a more realistic measure of effective cytotoxic exposure. Because of its effect on peak concentration, MISO reduces these values to an extent which depends on the minimum effective concentration (MEC) chosen (Table III). It can be seen that the lower the MEC is set the smaller the reduction of AUC becomes. For example, the plasma nitrosourea AUC was reduced by factors of 1.13 and 9.6 for MEC values of 1 and $2\,\mu g\,ml^{-1}$ respectively.

It is significant that with MISO the early peak (Figure 4) was composed largely of CCNU, the plasma concentration being $3.1 \pm 1.2\,\mu g\,ml^{-1}$ (s.d.), which represents 50–60% of the peak total nitrosoureas, whereas in the controls the value was $0.46 \pm 0.05\,\mu g\,ml^{-1}$ which was only ~5% of the peak.

### Discussion

Although it has been shown previously that MISO can protect against the *in vitro* cytotoxicity of adriamycin and m-AMSA (West *et al.*, 1981; Twentyman, 1982), as far as we are aware this represents the first report of chemoprotection by MISO *in vivo*.

We have shown that MISO can clearly have two directly opposing effects on the activity of CCNU, depending on the route of administration of the cytotoxic agent. For i.p. CCNU, MISO has a sensitizing effect on tumour with little change in normal tissue response. On the other hand, MISO is protective when CCNU is given orally. The degree of protection appears to be similar in

**Table III** The dependence of the reduction of nitrosourea AUC by MISO on minimum effective nitrosourea concentration

| *Minimum effective concentration* ($\mu g\,ml^{-1}$ *or* $\mu g\,g^{-1}$) | *Plasma AUC* ($\mu g\,min\,ml^{-1}$) *Control* | *MISO* | *Fold reduction factor* | *Tumour AUC* ($\mu g\,min\,g^{-1}$) *Control* | *MISO* | *Fold reduction factor* |
|---|---|---|---|---|---|---|
| 0 0 | 705 | 662 | 1.06 | 451 | 410 | 1.1 |
| 0.5 | 630 | 588 | 1.07 | 423 | 389 | 1.09 |
| 0.8 | 618 | 559 | 1.11 | 387 | 225 | 1.72 |
| 1.0 | 589 | 520 | 1.13 | 358 | 55 | 7.0 |
| 1.5 | 448 | 320 | 1.40 | 321 | 40 | 8.0 |
| 2.0 | 460 | 48 | 9.6 | 273 | 35 | 7.8 |

normal and tumour tissue, with if anything a slightly greater protection in the tumour. It should be pointed out that these effects occur at a dose of MISO ($500\,mg\,kg^{-1}$ or $2.5\,mmol\,kg^{-1}$) which causes no change in mouse body temperature.

We previously showed that MISO reduces the clearance of i.p. CCNU, probably through inhibition of hepatic drug metabolising enzymes. In support of this mechanism we have shown that the same dose of MISO does inhibit drug metabolizing enzymes in mice (Workman *et al.*, 1983) and more recently that pharmacological concentrations of MISO are able to inhibit the hydroxylation of CCNU by mouse liver microsomes (Lee & Workman, 1984*b*). By overcoming the lag in drug penetration into the tumour this reduced clearance leads to a selective increase in peak nitrosourea concentration in the tumour but not in normal tissue, thus providing a pharmacokinetic mechanism for chemosensitization and therapeutic gain (Lee & Workman, 1983; 1984*a*; 1984*b*). Furthermore, detailed studies of the effect of MISO on tumour and normal tissue CCNU pharmacokinetics suggested that peak nitrosourea concentration and/or "early" AUC above a minimum effective concentration are the principle determinants of cytotoxicity (Lee & Workman, 1984*a*; Lee *et al.*, In press). The present paper shows that when CCNU is given orally, misonidazole reduces the peak nitrosourea concentration and "early" AUC, but not total AUC ($AUC_{0-\infty}$), both in the plasma and the tumour, and we propose this to be the underlying mechanism for the reduction of oral CCNU cytotoxicity.

Comparing the pharmacokinetic parameters of oral with i.p. CCNU in the absence of MISO it is clear that although parent CCNU levels are actually lower following oral administration, both the total nitrosourea peak concentration and "early" AUC in the tumour are greater because of the higher concentration of metabolites. This finding explains the comparatively greater tumour response we have obtained for oral CCNU, particularly since the metabolites may be more active than the parent compound (Wheeler *et al.*, 1977). The difference in peak concentration may be at least partly due to the fact that absorption is slower by the oral route and the liver is able to convert more completely the parent CCNU to monohydroxylated metabolites. Since these metabolites are much more hydrophilic than the parent compound (Wheeler *et al.*, 1977), they are likely to have lower apparent volumes of distribution and therefore be present at higher levels in the plasma. However, penetration into tumour tissue is clearly not impaired.

In contrast to tumour response we cannot explain the difference in acute lethal dose between oral and i.p. CCNU in terms of the altered plasma pharmacokinetics. At first sight, the higher plasma exposure might be expected to result in greater toxicity for the oral route whereas oral CCNU was in fact $\sim 1.5$ times less toxic. However two points should be borne in mind here. Firstly, deaths from oral CCNU tended to occur later (median 10 days) than the normal 4–7 days for i.p. CCNU, possibly suggesting a change in the dose-limiting organ, e.g. bone marrow instead of gut. Secondly, measurements of plasma concentrations will not necessarily reveal changes in concentrations at target organs, especially those at the site of uptake. We have previously found very high nitrosourea concentrations in the small intestine following i.p. CCNU (Lee & Workman, 1984*a*) and it may be that these are reduced when the drug is given orally. Further studies using specific assays for gut and bone marrow damage should allow the elucidation of the mechanism involved.

The effect of MISO on oral CCNU pharmacokinetics is very complex. However the considerably higher peak levels of CCNU in the presence of MISO is a clear indication that the drug metabolising process is impaired by the sensitizer. Rapid distribution of the parent drug that has escaped "first-pass" metabolism would then explain the transient nature of the peak. The persistent plateau region of the total nitrosourea elimination profile, which immediately follows the initial peak, is made up almost entirely of the hydroxylated metabolites. The steady-state situation suggests that the rate of formation of the metabolites is equalled by their rate of clearance, the latter also being slowed by MISO as with the i.p. route (Lee & Workman, 1983). It is significant that the clearance of total nitrosoureas returns to control level by $\sim 6\,h$. In previous experiments where i.p. CCNU was given at different intervals following the same dose of MISO we showed that the duration of pharmacokinetic modification was also about 6 h, and this reflects the time taken for the circulating MISO to fall below the minimum effective concentrations of about $100–300\,\mu g\,ml^{-1}$ (Workman, 1980; Lee & Workman, 1983).

Whatever the precise mechanism of these major changes in pharmacokinetics, it is certain that the large reduction by MISO in both the plasma and tumour peak nitrosourea concentrations and "early" AUC, but not $AUC_{0-\infty}$, is the likely reason for the apparent reduction of CCNU cytotoxicity. Furthermore, this lends support to our proposal that $AUC_{0-\infty}$ is comparatively less important than the other two parameters in determining the activity of nitrosoureas (Lee & Workman, 1984*a*).

We should be extremely cautious when speculating on the possible clinical consequences of these findings. Not only have we shown here that the pharmacokinetics are different for the oral compared to the i.p. route in mice, and that this results in altered drug efficacy, toxicity and therapeutic ratio, but our recent clinical studies have also shown that the pharmacokinetics of oral CCNU in man exhibit additional differences from those with either route in the mouse (Lee *et al.*, In press). What we can say is that MISO is able to act either as a chemosensitizer or as a chemoprotector depending on the nature of the alteration in CCNU pharmacokinetics. Furthermore, we know that these effects obtained with a relatively high dose MISO are not exclusively mouse phenomena, since equiactive doses of the more potent chemosensitizer benznidazole are well tolerated in patients (Roberts *et al.*, in press) and do produce marked changes in nitrosourea pharmacokinetics after oral CCNU (Roberts *et al.*, in preparation). We therefore strongly recommend that the use of such regimes in patients should be accompanied by detailed pharmacokinetic investigations.

We are grateful to Prof. N.M. Bleehen for his support. We also wish to thank Dr C.E. Smithen of Roche Products Ltd. (Welwyn) for the supplies of misonidazole; Dr T.P. Johnson of the Southern Research Institute (Alabama, U.S.A.) for the synthetic CCNU metabolites; Lundbeck and Dr. Ven Narayanan of the N.C.I. for CCNU. Thanks are also due to Jane Donaldson for excellent technical assistance.

### References

CLUTTERBUCK, R.D., MILLAR, J.L. & McELWAIN, T.J. (1982). Misonidazole enhancement of the action of BCNU and melphalan against human melanoma xenografts *Am. J. Clin. Oncol.*, **5**, 73.

COLEMAN, C.N., FRIEDMAN, M.K., JACOBS, C. & 7 others. (1983). Phase I trial of intravenous L-phenylalanine mustard plus the sensitizer misonidazole. *Cancer Res.*, **43**, 5022.

HINCHLIFFE, M., McNALLY, N.J. & STRATFORD, M.R.L. (1983). The effect of radiosensitizers on the pharmacokinetics of melphalan and cyclophosphamide in the mouse. *Br. J. Cancer*, **48**, 375.

HIRST, D.G., BROWN, J.M. & HAZLEHURST, J.L. (1982). Enhancement of CCNU cytotoxicity by misonidazole: possible therapeutic gain. *Br. J. Cancer*, **46**, 109.

LEE, F.Y.F. & WORKMAN, P. (1983). Modification of CCNU pharmacokinetics by misonidazole – a major mechanism of chemosensitisation in mice. *Br. J. Cancer*, **47**, 659.

LEE, F.Y.F. & WORKMAN, P. (1984*a*). Misonidazole and CCNU: Further evidence for a pharmacokinetic mechanism of chemosensitization and therapeutic gain. *Br. J. Cancer*, **49**, 579.

LEE, F.Y.F. & WORKMAN, P. (1984*b*). Inhibition of liver microsomal metabolism of CCNU by nitroimidazoles – correlation with chemosensitization activity. *Br. J. Cancer*, (in press).

LEE, F., WORKMAN, P., ROBERTS, J.T. & BLEEHEN, N.M. (1985). Clinical pharmacokinetics of oral CCNU (Lomustine). *Cancer Chemother. Pharmacol.* (in press).

McNALLY, N.J. (1982). Enhancement of chemotherapy agents. *Int. J. Radiat. Oncol. Biol. Phys.*, **8**, 593.

ROBERTS, J.T., BLEEHEN, N.M., LEE, F.Y.F., WORKMAN, P. & WALTON, M.I. (1984). A phase 1 study of the combination of benznidazole and CCNU in man. *Int. J. Radiat. Oncol. Biol. Phys.*, **10**, 1745

SIEMANN, D.W. (1981). *In vivo* combination of misonidazole and the chemotherapeutic agent CCNU. *Br. J. Cancer*, **43**, 367.

SIEMANN, D.W. (1982). Response of murine tumours to combinations of CCNU with misonidazole and other radiation sensitizers. *Br. J. Cancer*, **45**, 272.

SIEMANN, D.W. (1984). Modification of chemotherapy by nitroimidazoles. *Int. J. Radiat. Oncol. Biol. Phys.* **10**, 1585.

SKIPPER, H.E., SCHABEL, F.M., Jr., MELLETT, L.B. & 4 others. (1970). Implications of biochemical, cytokinetic, pharmacologic, and toxicologic relationships in the design of optimal therapeutic schedules. *Cancer Chemother. Rep.*, **54**, 431.

TANNOCK, I.F. (1980). *In vivo* interaction of anticancer drugs with misonidazole or metronidazole: Cyclophosphamide and BCNU. *Br. J. Cancer*, **42**, 871.

TWENTYMAN, P.R. (1982). Growth delay in small EMT6 spheroids induced by cytotoxic drugs and its modification by misonidazole pretreatment under hypoxic condition. *Br. J. Cancer*, **45**, 565.

TWENTYMAN, P.R., KALLMAN, R.F. & BROWN, J.M. (1979). The effect of time between X-irradiation and chemotherapy on the growth of three solid mouse tumours – I. Adriamycin. *Int. J. Radiat. Oncol. Biol. Phys.*, **5**, 1255.

WEST, C., STRATFORD, I.J., BARRASS, N. & SMITH, E. (1981). A comparison of adriamycin and mAMSA *in vitro*: Cell lethality and SCE studies. *Br. J. Cancer*, **44**, 798.

WHEELER, G.P., JOHNSTON, T.P., BOWDON, B.J., McCALEB, G.S., HILL, D.L. & MONTGOMERY, J.A. (1977). Comparison of the properties of metabolites of CCNU. *Biochem. Pharmacol.*, **26**, 2331.

WORKMAN, P. (1980). Dose-dependence and related studies on the pharmacokinetics of misonidazole and desmethylmisonidazole in mice. *Cancer Chemother. Pharmacol.*, **5**, 27.

WORKMAN, P. & TWENTYMAN, P.R. (1982). Structure/activity relationships for the enhancement by electron-affinic drugs of the anti-tumour effect of CCNU. *Br. J. Cancer*, **46**, 249.

WORKMAN, P., TWENTYMAN, P.R., LEE, F.Y.F. & WALTON, M. (1983). Drug metabolism and chemosensitisation: nitroimidazoles as inhibitors of drug metabolism. *Biochem. Pharmacol.*, **32**, 857.